# Supplementary material for: Multi‐dataset identification of innovative feature genes and molecular mechanisms in keratoconus
Source: J Cell Mol Med. 2024 Sep 19;28(18):e70079. doi: 10.1111/jcmm.70079 (PMC11412914; doi:10.1111/jcmm.70079)
Supplement: Supplementary file 2 — Material S2. [file JCMM-28-e70079-s002.zip › 7.GSEA analysis/5.RNF217-AS1/KEGG_NEUROACTIVE_LIGAND_RECEPTOR_INTERACTION.html]

Details for gene set KEGG\_NEUROACTIVE\_LIGAND\_RECEPTOR\_INTERACTION[GSEA]

|  || Dataset | RNF217-AS1.group.cls#h\_versus\_l |
| Phenotype | group.cls#h\_versus\_l |
| Upregulated in class | h |
| GeneSet | KEGG\_NEUROACTIVE\_LIGAND\_RECEPTOR\_INTERACTION |
| Enrichment Score (ES) | 0.24971795 |
| Normalized Enrichment Score (NES) | 0.9046954 |
| Nominal p-value | 0.78571427 |
| FDR q-value | 1.0 |
| FWER p-Value | 1.0 |
Table: GSEA Results Summary

  

Fig 1: Enrichment plot: KEGG\_NEUROACTIVE\_LIGAND\_RECEPTOR\_INTERACTION      
 Profile of the Running ES Score & Positions of GeneSet Members on the Rank Ordered List

  

| SYMBOL | TITLE | RANK IN GENE LIST | RANK METRIC SCORE | RUNNING ES | CORE ENRICHMENT || 1 | TSHR | na | 64 | 0.807 | 0.0152 | Yes |
| 2 | TRHR | na | 147 | 0.691 | 0.0275 | Yes |
| 3 | CHRNB2 | na | 379 | 0.565 | 0.0328 | Yes |
| 4 | NPY2R | na | 386 | 0.563 | 0.0445 | Yes |
| 5 | TRPV1 | na | 477 | 0.537 | 0.0533 | Yes |
| 6 | GRIK2 | na | 553 | 0.519 | 0.0621 | Yes |
| 7 | DRD5 | na | 593 | 0.511 | 0.0718 | Yes |
| 8 | DRD3 | na | 780 | 0.473 | 0.0764 | Yes |
| 9 | CHRNE | na | 790 | 0.471 | 0.0861 | Yes |
| 10 | ADRA1D | na | 846 | 0.463 | 0.0943 | Yes |
| 11 | PTGIR | na | 1200 | 0.419 | 0.0930 | Yes |
| 12 | GLP1R | na | 1251 | 0.414 | 0.1003 | Yes |
| 13 | GABRR2 | na | 1352 | 0.405 | 0.1060 | Yes |
| 14 | GRID1 | na | 1356 | 0.405 | 0.1145 | Yes |
| 15 | P2RY13 | na | 1418 | 0.400 | 0.1212 | Yes |
| 16 | GABRG3 | na | 1484 | 0.394 | 0.1276 | Yes |
| 17 | MLNR | na | 1548 | 0.387 | 0.1340 | Yes |
| 18 | GRIN3B | na | 1655 | 0.378 | 0.1390 | Yes |
| 19 | OPRM1 | na | 1818 | 0.365 | 0.1420 | Yes |
| 20 | PRSS3 | na | 1978 | 0.352 | 0.1448 | Yes |
| 21 | ADORA1 | na | 2014 | 0.349 | 0.1512 | Yes |
| 22 | HTR2C | na | 2142 | 0.339 | 0.1547 | Yes |
| 23 | HTR6 | na | 2244 | 0.331 | 0.1588 | Yes |
| 24 | GABBR1 | na | 2604 | 0.310 | 0.1549 | Yes |
| 25 | CCKBR | na | 2652 | 0.308 | 0.1601 | Yes |
| 26 | GABRA2 | na | 2692 | 0.306 | 0.1654 | Yes |
| 27 | GLRA1 | na | 2738 | 0.304 | 0.1706 | Yes |
| 28 | CGA | na | 2795 | 0.300 | 0.1753 | Yes |
| 29 | HTR1A | na | 3022 | 0.290 | 0.1749 | Yes |
| 30 | OXTR | na | 3035 | 0.290 | 0.1807 | Yes |
| 31 | SSTR1 | na | 3151 | 0.284 | 0.1834 | Yes |
| 32 | GABRG1 | na | 3184 | 0.283 | 0.1885 | Yes |
| 33 | GRM5 | na | 3284 | 0.279 | 0.1915 | Yes |
| 34 | RXFP1 | na | 3474 | 0.272 | 0.1918 | Yes |
| 35 | F2RL1 | na | 3581 | 0.268 | 0.1944 | Yes |
| 36 | GABRA3 | na | 3659 | 0.265 | 0.1978 | Yes |
| 37 | FSHB | na | 3675 | 0.264 | 0.2029 | Yes |
| 38 | AGTR1 | na | 3697 | 0.263 | 0.2079 | Yes |
| 39 | ADRA2B | na | 3698 | 0.263 | 0.2135 | Yes |
| 40 | GRIK1 | na | 3871 | 0.257 | 0.2140 | Yes |
| 41 | CHRNA2 | na | 3891 | 0.257 | 0.2188 | Yes |
| 42 | P2RX6 | na | 4024 | 0.251 | 0.2203 | Yes |
| 43 | OPRK1 | na | 4171 | 0.246 | 0.2213 | Yes |
| 44 | P2RY10 | na | 4334 | 0.240 | 0.2217 | Yes |
| 45 | GRIN2C | na | 4492 | 0.234 | 0.2221 | Yes |
| 46 | GNRHR | na | 4497 | 0.234 | 0.2270 | Yes |
| 47 | CHRNA1 | na | 4525 | 0.233 | 0.2311 | Yes |
| 48 | GRM4 | na | 4576 | 0.231 | 0.2346 | Yes |
| 49 | GPR156 | na | 4800 | 0.224 | 0.2328 | Yes |
| 50 | C5AR1 | na | 5106 | 0.213 | 0.2285 | Yes |
| 51 | GALR2 | na | 5115 | 0.213 | 0.2328 | Yes |
| 52 | LEP | na | 5215 | 0.209 | 0.2343 | Yes |
| 53 | AGTR2 | na | 5655 | 0.194 | 0.2257 | Yes |
| 54 | MC1R | na | 5728 | 0.192 | 0.2277 | Yes |
| 55 | BDKRB2 | na | 5730 | 0.192 | 0.2317 | Yes |
| 56 | KISS1R | na | 5823 | 0.189 | 0.2330 | Yes |
| 57 | GALR1 | na | 5860 | 0.187 | 0.2360 | Yes |
| 58 | SSTR4 | na | 5939 | 0.185 | 0.2376 | Yes |
| 59 | SCTR | na | 6111 | 0.180 | 0.2365 | Yes |
| 60 | PTGER3 | na | 6298 | 0.174 | 0.2348 | Yes |
| 61 | CNR1 | na | 6521 | 0.167 | 0.2319 | Yes |
| 62 | CHRNB4 | na | 6537 | 0.167 | 0.2350 | Yes |
| 63 | RXFP2 | na | 6614 | 0.164 | 0.2362 | Yes |
| 64 | PRLR | na | 6689 | 0.162 | 0.2375 | Yes |
| 65 | CHRM5 | na | 6728 | 0.161 | 0.2398 | Yes |
| 66 | GH1 | na | 6929 | 0.155 | 0.2373 | Yes |
| 67 | SSTR3 | na | 6953 | 0.154 | 0.2399 | Yes |
| 68 | THRB | na | 7000 | 0.153 | 0.2418 | Yes |
| 69 | P2RY4 | na | 7006 | 0.153 | 0.2449 | Yes |
| 70 | NPY4R | na | 7080 | 0.151 | 0.2460 | Yes |
| 71 | P2RY6 | na | 7438 | 0.141 | 0.2386 | Yes |
| 72 | TAAR2 | na | 7466 | 0.141 | 0.2408 | Yes |
| 73 | ADRA1B | na | 7589 | 0.138 | 0.2402 | Yes |
| 74 | HCRTR1 | na | 7892 | 0.130 | 0.2342 | Yes |
| 75 | GIPR | na | 7953 | 0.129 | 0.2352 | Yes |
| 76 | ADRA2A | na | 7968 | 0.129 | 0.2375 | Yes |
| 77 | NPFFR2 | na | 7985 | 0.128 | 0.2398 | Yes |
| 78 | S1PR3 | na | 8016 | 0.127 | 0.2416 | Yes |
| 79 | PTGFR | na | 8023 | 0.127 | 0.2441 | Yes |
| 80 | CYSLTR2 | na | 8030 | 0.127 | 0.2466 | Yes |
| 81 | HTR4 | na | 8079 | 0.126 | 0.2479 | Yes |
| 82 | SSTR2 | na | 8109 | 0.125 | 0.2497 | Yes |
| 83 | P2RY2 | na | 8453 | 0.117 | 0.2422 | No |
| 84 | CHRND | na | 8490 | 0.116 | 0.2437 | No |
| 85 | CHRNA10 | na | 8523 | 0.116 | 0.2452 | No |
| 86 | F2RL3 | na | 8700 | 0.112 | 0.2425 | No |
| 87 | PTH2R | na | 8851 | 0.108 | 0.2404 | No |
| 88 | ADORA2B | na | 8864 | 0.108 | 0.2423 | No |
| 89 | NTSR1 | na | 8996 | 0.105 | 0.2408 | No |
| 90 | APLNR | na | 9389 | 0.097 | 0.2314 | No |
| 91 | GRIN2A | na | 9393 | 0.097 | 0.2334 | No |
| 92 | CTSG | na | 9428 | 0.097 | 0.2345 | No |
| 93 | GLRB | na | 9909 | 0.088 | 0.2224 | No |
| 94 | TSHB | na | 10024 | 0.086 | 0.2209 | No |
| 95 | LPAR3 | na | 10177 | 0.083 | 0.2183 | No |
| 96 | C3AR1 | na | 10261 | 0.082 | 0.2176 | No |
| 97 | TAAR5 | na | 10364 | 0.080 | 0.2164 | No |
| 98 | CHRNA9 | na | 10621 | 0.076 | 0.2105 | No |
| 99 | HRH3 | na | 10651 | 0.075 | 0.2113 | No |
| 100 | OPRD1 | na | 10736 | 0.074 | 0.2104 | No |
| 101 | NTSR2 | na | 10956 | 0.071 | 0.2056 | No |
| 102 | PTGER1 | na | 11033 | 0.069 | 0.2048 | No |
| 103 | BRS3 | na | 11135 | 0.068 | 0.2033 | No |
| 104 | PARD3 | na | 11290 | 0.066 | 0.2003 | No |
| 105 | CCKAR | na | 11307 | 0.066 | 0.2012 | No |
| 106 | GCGR | na | 11373 | 0.065 | 0.2007 | No |
| 107 | GRIK4 | na | 11523 | 0.062 | 0.1977 | No |
| 108 | GABRA6 | na | 11617 | 0.061 | 0.1963 | No |
| 109 | GRIN3A | na | 11723 | 0.059 | 0.1945 | No |
| 110 | PRSS1 | na | 11768 | 0.058 | 0.1944 | No |
| 111 | GRM6 | na | 12114 | 0.054 | 0.1856 | No |
| 112 | TAAR9 | na | 12141 | 0.053 | 0.1860 | No |
| 113 | GABRA1 | na | 12159 | 0.053 | 0.1866 | No |
| 114 | GABRA4 | na | 12178 | 0.053 | 0.1872 | No |
| 115 | TAAR1 | na | 12188 | 0.053 | 0.1880 | No |
| 116 | PTAFR | na | 12213 | 0.053 | 0.1885 | No |
| 117 | GPR35 | na | 12287 | 0.052 | 0.1874 | No |
| 118 | FPR2 | na | 12396 | 0.050 | 0.1854 | No |
| 119 | HTR2B | na | 12943 | 0.043 | 0.1704 | No |
| 120 | GABRB2 | na | 13070 | 0.041 | 0.1677 | No |
| 121 | HTR1B | na | 13075 | 0.041 | 0.1684 | No |
| 122 | GH2 | na | 13130 | 0.040 | 0.1677 | No |
| 123 | GABRR1 | na | 13217 | 0.039 | 0.1660 | No |
| 124 | LHB | na | 13286 | 0.039 | 0.1649 | No |
| 125 | TAAR6 | na | 13500 | 0.036 | 0.1595 | No |
| 126 | GHR | na | 13579 | 0.035 | 0.1580 | No |
| 127 | CHRNA4 | na | 13644 | 0.035 | 0.1568 | No |
| 128 | F2RL2 | na | 14132 | 0.029 | 0.1433 | No |
| 129 | MAS1 | na | 14359 | 0.026 | 0.1373 | No |
| 130 | GRID2 | na | 14601 | 0.023 | 0.1308 | No |
| 131 | PRLHR | na | 14642 | 0.023 | 0.1301 | No |
| 132 | HTR1F | na | 14968 | 0.019 | 0.1211 | No |
| 133 | LTB4R2 | na | 15075 | 0.018 | 0.1184 | No |
| 134 | P2RX4 | na | 15617 | 0.013 | 0.1030 | No |
| 135 | LPAR4 | na | 15897 | 0.010 | 0.0951 | No |
| 136 | CHRNB1 | na | 15947 | 0.009 | 0.0939 | No |
| 137 | CHRNA3 | na | 16023 | 0.008 | 0.0919 | No |
| 138 | TACR2 | na | 16054 | 0.008 | 0.0912 | No |
| 139 | TAAR8 | na | 16086 | 0.008 | 0.0905 | No |
| 140 | GLRA2 | na | 16128 | 0.007 | 0.0894 | No |
| 141 | HTR1E | na | 16224 | 0.007 | 0.0868 | No |
| 142 | BDKRB1 | na | 17163 | -0.003 | 0.0597 | No |
| 143 | FSHR | na | 17407 | -0.006 | 0.0527 | No |
| 144 | GRIK5 | na | 17520 | -0.007 | 0.0496 | No |
| 145 | HCRTR2 | na | 17763 | -0.009 | 0.0428 | No |
| 146 | TSPO | na | 17802 | -0.009 | 0.0419 | No |
| 147 | PLG | na | 17835 | -0.009 | 0.0411 | No |
| 148 | NPY1R | na | 17931 | -0.010 | 0.0386 | No |
| 149 | PRL | na | 18227 | -0.013 | 0.0303 | No |
| 150 | TACR1 | na | 18235 | -0.013 | 0.0304 | No |
| 151 | F2 | na | 18355 | -0.014 | 0.0272 | No |
| 152 | ADRA1A | na | 18508 | -0.016 | 0.0231 | No |
| 153 | GRM7 | na | 18565 | -0.016 | 0.0219 | No |
| 154 | GRM2 | na | 18751 | -0.018 | 0.0169 | No |
| 155 | ADRA2C | na | 19119 | -0.021 | 0.0067 | No |
| 156 | ADRB2 | na | 19191 | -0.022 | 0.0051 | No |
| 157 | LHCGR | na | 19494 | -0.025 | -0.0032 | No |
| 158 | AVPR1B | na | 19611 | -0.026 | -0.0060 | No |
| 159 | S1PR1 | na | 19668 | -0.027 | -0.0070 | No |
| 160 | LTB4R | na | 19762 | -0.028 | -0.0091 | No |
| 161 | GHSR | na | 19937 | -0.029 | -0.0136 | No |
| 162 | LPAR6 | na | 20205 | -0.032 | -0.0206 | No |
| 163 | P2RX2 | na | 20289 | -0.033 | -0.0224 | No |
| 164 | TBXA2R | na | 20417 | -0.034 | -0.0253 | No |
| 165 | CSH1 | na | 20697 | -0.037 | -0.0326 | No |
| 166 | GRIA2 | na | 20870 | -0.039 | -0.0368 | No |
| 167 | MC5R | na | 21260 | -0.043 | -0.0472 | No |
| 168 | GRPR | na | 21358 | -0.044 | -0.0491 | No |
| 169 | ADRB1 | na | 21391 | -0.044 | -0.0491 | No |
| 170 | HTR1D | na | 21559 | -0.046 | -0.0530 | No |
| 171 | S1PR2 | na | 21612 | -0.046 | -0.0535 | No |
| 172 | ADCYAP1R1 | na | 21659 | -0.047 | -0.0538 | No |
| 173 | NR3C1 | na | 21752 | -0.048 | -0.0555 | No |
| 174 | P2RY14 | na | 21851 | -0.049 | -0.0573 | No |
| 175 | DRD4 | na | 21899 | -0.050 | -0.0576 | No |
| 176 | DRD1 | na | 22536 | -0.056 | -0.0748 | No |
| 177 | S1PR4 | na | 22541 | -0.057 | -0.0738 | No |
| 178 | NMBR | na | 22557 | -0.057 | -0.0730 | No |
| 179 | CRHR2 | na | 22826 | -0.060 | -0.0795 | No |
| 180 | CHRNA6 | na | 22832 | -0.060 | -0.0784 | No |
| 181 | P2RX3 | na | 23635 | -0.070 | -0.1002 | No |
| 182 | MTNR1A | na | 23732 | -0.071 | -0.1014 | No |
| 183 | AVPR2 | na | 23857 | -0.073 | -0.1035 | No |
| 184 | CNR2 | na | 23930 | -0.074 | -0.1040 | No |
| 185 | FPR3 | na | 23997 | -0.075 | -0.1043 | No |
| 186 | GABRD | na | 24173 | -0.078 | -0.1078 | No |
| 187 | GALR3 | na | 24263 | -0.079 | -0.1087 | No |
| 188 | HRH1 | na | 24273 | -0.079 | -0.1073 | No |
| 189 | MC3R | na | 24308 | -0.080 | -0.1066 | No |
| 190 | VIPR2 | na | 24366 | -0.081 | -0.1065 | No |
| 191 | GRIN1 | na | 24441 | -0.082 | -0.1069 | No |
| 192 | EDNRB | na | 24896 | -0.089 | -0.1182 | No |
| 193 | P2RY1 | na | 24948 | -0.089 | -0.1178 | No |
| 194 | LPAR2 | na | 24999 | -0.090 | -0.1173 | No |
| 195 | HTR5A | na | 25386 | -0.097 | -0.1265 | No |
| 196 | LPAR1 | na | 25719 | -0.102 | -0.1340 | No |
| 197 | PTH1R | na | 26122 | -0.109 | -0.1433 | No |
| 198 | NMUR1 | na | 26249 | -0.111 | -0.1446 | No |
| 199 | UTS2R | na | 26412 | -0.115 | -0.1469 | No |
| 200 | GRIN2B | na | 26448 | -0.115 | -0.1454 | No |
| 201 | DRD2 | na | 26679 | -0.119 | -0.1496 | No |
| 202 | LEPR | na | 26856 | -0.123 | -0.1521 | No |
| 203 | S1PR5 | na | 26898 | -0.123 | -0.1507 | No |
| 204 | GLP2R | na | 26917 | -0.124 | -0.1486 | No |
| 205 | GABRE | na | 27040 | -0.127 | -0.1494 | No |
| 206 | HRH2 | na | 27361 | -0.133 | -0.1559 | No |
| 207 | CALCRL | na | 27864 | -0.145 | -0.1674 | No |
| 208 | GHRHR | na | 27975 | -0.148 | -0.1674 | No |
| 209 | PTGER2 | na | 28050 | -0.150 | -0.1664 | No |
| 210 | GABRG2 | na | 28073 | -0.150 | -0.1638 | No |
| 211 | GABRB3 | na | 28130 | -0.152 | -0.1623 | No |
| 212 | GRM1 | na | 28181 | -0.153 | -0.1605 | No |
| 213 | CHRM4 | na | 28246 | -0.155 | -0.1590 | No |
| 214 | NPBWR2 | na | 28253 | -0.155 | -0.1559 | No |
| 215 | TACR3 | na | 28351 | -0.157 | -0.1554 | No |
| 216 | HTR2A | na | 28374 | -0.158 | -0.1527 | No |
| 217 | CRHR1 | na | 28381 | -0.158 | -0.1495 | No |
| 218 | ADORA3 | na | 28389 | -0.158 | -0.1464 | No |
| 219 | CHRM1 | na | 28575 | -0.164 | -0.1482 | No |
| 220 | THRA | na | 28589 | -0.165 | -0.1451 | No |
| 221 | MC4R | na | 28612 | -0.165 | -0.1423 | No |
| 222 | CHRM3 | na | 28675 | -0.167 | -0.1405 | No |
| 223 | EDNRA | na | 28814 | -0.171 | -0.1409 | No |
| 224 | GABBR2 | na | 28874 | -0.173 | -0.1390 | No |
| 225 | GRIN2D | na | 29412 | -0.188 | -0.1505 | No |
| 226 | CHRNA5 | na | 29503 | -0.191 | -0.1491 | No |
| 227 | GRIA3 | na | 29565 | -0.193 | -0.1468 | No |
| 228 | CHRNG | na | 29666 | -0.196 | -0.1455 | No |
| 229 | AVPR1A | na | 29821 | -0.201 | -0.1458 | No |
| 230 | ADORA2A | na | 30043 | -0.208 | -0.1478 | No |
| 231 | F2R | na | 30147 | -0.211 | -0.1463 | No |
| 232 | GABRB1 | na | 30254 | -0.215 | -0.1448 | No |
| 233 | GRIA4 | na | 30995 | -0.242 | -0.1611 | No |
| 234 | P2RX1 | na | 31065 | -0.245 | -0.1580 | No |
| 235 | GZMA | na | 31122 | -0.247 | -0.1544 | No |
| 236 | GRIA1 | na | 31197 | -0.250 | -0.1512 | No |
| 237 | HTR7 | na | 31228 | -0.251 | -0.1468 | No |
| 238 | CHRM2 | na | 31259 | -0.252 | -0.1423 | No |
| 239 | CHRNA7 | na | 31346 | -0.256 | -0.1393 | No |
| 240 | ADRB3 | na | 31485 | -0.263 | -0.1378 | No |
| 241 | GLRA3 | na | 31517 | -0.264 | -0.1331 | No |
| 242 | OPRL1 | na | 31535 | -0.265 | -0.1279 | No |
| 243 | CHRNB3 | na | 31541 | -0.266 | -0.1225 | No |
| 244 | VIPR1 | na | 31591 | -0.268 | -0.1182 | No |
| 245 | HRH4 | na | 31631 | -0.270 | -0.1136 | No |
| 246 | FPR1 | na | 31916 | -0.286 | -0.1158 | No |
| 247 | NPY5R | na | 31918 | -0.287 | -0.1097 | No |
| 248 | GRM3 | na | 31995 | -0.291 | -0.1058 | No |
| 249 | NPBWR1 | na | 32074 | -0.296 | -0.1018 | No |
| 250 | P2RX5 | na | 32137 | -0.299 | -0.0972 | No |
| 251 | GRM8 | na | 32319 | -0.311 | -0.0959 | No |
| 252 | NPFFR1 | na | 32457 | -0.321 | -0.0931 | No |
| 253 | GPR50 | na | 32678 | -0.336 | -0.0923 | No |
| 254 | SSTR5 | na | 32747 | -0.342 | -0.0871 | No |
| 255 | MCHR1 | na | 32765 | -0.343 | -0.0803 | No |
| 256 | P2RY8 | na | 32777 | -0.344 | -0.0733 | No |
| 257 | CALCR | na | 32788 | -0.345 | -0.0663 | No |
| 258 | GABRQ | na | 32845 | -0.349 | -0.0605 | No |
| 259 | MTNR1B | na | 32944 | -0.356 | -0.0558 | No |
| 260 | GABRP | na | 33250 | -0.380 | -0.0566 | No |
| 261 | NMUR2 | na | 33255 | -0.380 | -0.0487 | No |
| 262 | GPR83 | na | 33350 | -0.390 | -0.0431 | No |
| 263 | MC2R | na | 33475 | -0.404 | -0.0382 | No |
| 264 | GABRA5 | na | 33668 | -0.428 | -0.0347 | No |
| 265 | P2RX7 | na | 33820 | -0.450 | -0.0295 | No |
| 266 | PTGER4 | na | 33901 | -0.463 | -0.0220 | No |
| 267 | PTGDR | na | 34043 | -0.490 | -0.0157 | No |
| 268 | CYSLTR1 | na | 34253 | -0.543 | -0.0103 | No |
| 269 | MCHR2 | na | 34319 | -0.564 | -0.0002 | No |
| 270 | GRIK3 | na | 34331 | -0.570 | 0.0115 | No |
Table: GSEA details [plain text format]

  

Fig 2: KEGG\_NEUROACTIVE\_LIGAND\_RECEPTOR\_INTERACTION      
 Blue-Pink O' Gram in the Space of the Analyzed GeneSet

  

Fig 3: KEGG\_NEUROACTIVE\_LIGAND\_RECEPTOR\_INTERACTION: Random ES distribution      
 Gene set null distribution of ES for **KEGG\_NEUROACTIVE\_LIGAND\_RECEPTOR\_INTERACTION**
